# Compliance with herpes zoster vaccination in young and adult individuals in two regions of Italy

**DOI:** 10.1186/1471-2458-10-333

**Published:** 2010-06-12

**Authors:** Antonino Parlato, Vincenzo Romano Spica, Massimo Ciccozzi, Francesca Farchi, Francesca Gallè, Valeria Di Onofrio, Elisabetta Franco, Giorgio Liguori

**Affiliations:** 1 Epidemiology and Prevention Departmental Area, Local Health Agency NA2, Via Corrado Alvaro 8, 80074, Pozzuoli (Naples), Italy; 2 Department of Health Sciences, University of Rome "Foro Italico", Piazza Lauro de Bosis 3, 00135, Rome, Italy; 3 Department of Infectious, Parasitic and Immune-Mediated Diseases, Istituto Superiore di Sanità (ISS), Viale Regina Elena 299, 00161, Rome, Italy; 4 Department of Studies of Institutions and Territorial Systems, University of Naples "Parthenope", Via Medina 40, 80133, Naples, Italy; 5 Department of Public Health, Faculty of Medicine, University of Rome "Tor Vergata", Via di Tor Vergata 135, 00173, Rome, Italy

## Abstract

**Background:**

The purpose of this work was to explore the knowledge and acceptance of Varicella Zoster Virus (VZV)-Herpes Zoster (HZ) vaccination in the general Italian population, where the HZ vaccine has not yet been distributed, using a prevalence study of subjects from two regions in Italy.

**Methods:**

A group of 3,173 individuals were interviewed using a questionnaire. The youngest age group (≤ 20 year) was composed of students interviewed at university. The middle age group (21-40 years) and the older age group (≥ 41 years) were interviewed by general practitioners in their office.

**Results:**

In both regions, the majority of subjects had been infected with varicella, and only 165 (5.2%) subjects reported receiving the VZV vaccination. Regarding HZ, 2,749 (86.6%) individuals stated that they knew of the virus and 2,233 (70%) were willing to be vaccinated against HZ. The majority of people willing to be vaccinated were in the middle and older age groups (36.6% and 44.7%, respectively).

**Conclusion:**

Compliance versus vaccination results were satisfactory and probably, with the upcoming availability of the HZ vaccine in Italy, adults will be favourably disposed towards vaccination.

## Background

Worldwide, varicella-zoster virus (VZV) infection (chickenpox) affects millions of individuals, causing significant suffering. During primary infection, the virus migrates to the dorsal root and cranial root ganglia, where it remains latent. Herpes zoster (HZ, shingles) is the result of reactivation of dormant VZV in the ganglia [1]. HZ causes acute and chronic morbidity and is characterized by a painful vesicular unilateral dermatomal rash. The most common HZ complication is post-herpetic neuralgia (PHN), which consists of burning pain, paresthesias, dysesthesia, pruritus, or anesthesia in the affected area and most frequently develops in subjects over 60 years of age. The risk of developing HZ in people exposed to VZV ranges from 10% to 30%, with an increased incidence in older individuals and in immunocompromised hosts [2-6]. Cell-mediate immunity (CMI) is thought to be important in defense against VZV infection. Therefore, a defective CMI is a major factor in virus reactivation [1].

It has been predicted that a quarter of the general population will be affected by HZV reactivation during their lifetime [7]. In the United States (US), an estimated one million cases of HZ infection occur each year, with an incidence of 11.12 cases per 1,000 patient-years and there are higher incidence rates and number of hospitalizations among older adults (> 50 years) [8-12]. Studies examining the general population of European countries demonstrated that the incidence of HZ in all ages ranges was from 1.2 to 4.8 cases per 1000 person-years and, among people > 60 years of age, it was 7.2 to 11.8 cases per 1,000 person-years [13,14]. Also, in a Spanish study, the majority of hospitalizations for HZ occurred in adults over 50 years of age [15]. In Italy, a regional survey conducted in 2007 reported a total incidence of 1.74 HZ cases/1000 population per year, over a period of 14 years and an incidence of hospital admissions of 0.12/1000 inhabitants, with an average cost of €4082.59 per hospitalization [16].

In 2006, an HZ vaccine was approved by the US Food and Drug Administration (US FDA) and recommended by the Advisory Committee on Immunization Practices (ACIP) for the prevention of shingles in individuals ≥ 60 years old. In the same year, European Medicines Agency (EMEA) gave its Marketing Authorization valid throughout the European Union to this product. EU approval states that the recommended age of vaccination starts from 50 yrs of age instead of 60 yrs of age as in the USA http://www.ema.europa.eu/humandocs/Humans/EPAR/zostavax/zostavax.htm. This new vaccine is made of the same live attenuated VZV strain used in the varicella vaccine, but it has a potency at least 14-times higher than that of single-antigen varicella vaccine, which is necessary to confer protection against zoster [17]. It boosts the CMI against HZ, thus reducing the incidence of the disease and severity [8,18-20]. Efficacy, safety and tolerability of a high titre VZV vaccine for the prevention of HZ infection and its sequelae were demonstrated in a multicentric study of over 38,000 older adults [9,10].

Nowadays, the vaccine is only distributed in USA; vaccination has been recommended in Austria and Switzerland and it should be soon included in European geriatric guidelines [21].

In Italy, the HZ vaccine is not yet commercially available, though, it is about to be distributed. However, few population-based studies have been performed in Italy assessing the subjective perception of the relevance of VZV to health and the importance of vaccination. The aim of this study was to explore the knowledge of VZV infections in the general population of two Italian regions and analyze the VZV vaccine immunization status and the acceptance of HZ vaccination.

## Methods

The sample group was composed by 3,173 subjects from two Italian Regions, Campania and Lazio. Subjects were consecutively enrolled: the youngest age group (≤ 20 year) was composed of university students interviewed by teachers; the middle age group (21-40 years) and the eldest age group (≥ 41 years) were interviewed by general practitioners in their office. Face-to-face interviews were done by administrating a brief questionnaire divided in two sections (Additional file 1).

The first section included general questions about sex, age, level of education, residence and nationality. In the second section, the subjects were asked about their knowledge of and personal experience with both chickenpox and shingles, and also, about their compliance versus HZ vaccination. Questionnaire was developed on the basis of previous experiences in collaboration with Sanofi Pasteur MSD and tested on a sample of university students. Statistical analysis was performed using the Epi Info software, version 3.32.

## Results

Of the 3,173 subjects interviewed, 1,469 (46.3%) were men. The median age for both men and women was 36 years, ranging from 16 to 92 years. Overall, 2,017 subjects (63.5%) reported higher educational levels, i.e., secondary school and university. Moreover, the sample was homogeneously distributed within the two regions (Table 1). In the population interviewed, 2,256 (71.1%) individuals claimed that they had had chickenpox during their lifetime (22%, 41.4% and 36.7% from the three age groups, respectively). Only 165 individuals (5.2%) reported being vaccinated for VZV. With regard to the knowledge of HZ, 2,749 individuals (86.6%) reported that they knew of the disease and, notably, 352 (11.1%) had had the disease in the past (Table 2). Of the 2,082 individuals who reported that they knew the severity of the disease, 1,528 subjects were 1.5 times more probable to accept the vaccination (OR 1.5, CI._95 _= 1.29-1.77, *p*-value = 1 × 10^-6^). Comparing financial statements, and using the educational level as a surrogate, people with higher educational levels (degree and secondary school) seem to be more likely to accept vaccination than those with a lower educational level (primary school) (OR 0.65, CI._95 _= 0.48-0.89, *p*-value < .05 compared to OR 0.63, CI._95 _= 0.90, *p*-value < .05, respectively). Moreover, individuals who have had VZV in the past are more likely to accept the vaccination than people with no previous history of VZV (OR 1.3, CI._95 _= 1.10-1.54, *p*-value = .001).

**Table 1:** Characteristics of subjects interviewed

	N.	%
**Area of residence:**		

Lazio	1,456	45.8
Campania	1,717	54.1

**Sex:**		

Females	1,704	53.7
Males	1,469	46.3

**Age:**		

<21	594	18.7
21-40	1,162	36.6
≥41	1,417	44.7

**Nationality:**		

Other	89	2.8
Italian	3,084	97.2

**Education:**		

None	152	4.8
Primary/Middle school	1,004	31.6
Secondary school	1,724	54.3
University	293	9.2

**Table 2:** Individuals who know or who were affected by VZV-associated diseases and their compliance with vaccinations

	**Varicella**			**HZ**		
	
**subjects** **(n. 3173)**	**knowing (n. 3071)**	**affected (n. 2256)**	**immunized (n. 165)**	**knowing (n. 2749)**	**affected (n. 352)**	**compliance (n. 2233)**
	
**young **N = 594	35.1%	22.0%	64.4%	28.4%	3.04%	18.7%
**adults **N = 1,162	33.5%	41.4%	28.5%	34.0%	41.2%	36.6%
**older **N = 1,417	31.4%	36.7%	9.09%	37.6%	55.7%	44.7%

Among the 3,173 individuals interviewed about their knowledge of HZ, with respect to the acceptance of the vaccination, 1,960 (61.77%) individuals claimed to be amenable towards this type of prevention because they were familiar with the disease (OR 1.4, CI._95 _= 1.17-1.82, *p*-value = 7 × 10^-4^). Furthermore, there were more people who were willing to be vaccinated in the middle and older adult age groups (36.6% and 44.7%, respectively) than in the youngest age class (OR 1.34, CI._95 _= 1.07-1.66, *p*-value = 7 × 10^-3^).

## Discussion

VZV can cause two clinical conditions, the first results from the primary infection and the second is due to reactivation of the virus following the initial infection. Both of these diseases are often associated with severe complications. In 1995, a varicella vaccination program was implemented in the United States [22]. The immediate goal of the extensive vaccination against VZV was to significantly decrease the morbidity and mortality associated with the disease. With widespread vaccination, there has been a concomitant decline in the incidence of the disease, along with a decrease in hospitalizations and deaths due to VZV infection [23]. To improve protection, a two-dose schedule of immunization was recommended for routine use in children by the Centers for Disease Control and Prevention (CDCs) in June 2006. At the same time, the licensure of the combined measles-mumps-rubella-varicella vaccine was completed, which allowed harmonization of immunization against these four viruses with one injection given twice in childhood [23,24].

The Shingles Prevention Study, a case-control study examining the widespread immunization against VZV, established that the zoster vaccine was safe, well-tolerated, and effective at reducing the burden of illness due to HZ and the incidence of PHN [9,10,25-27].

In 2006, the US FDA licensed the HZ vaccine [8], which promises to reduce the morbidity and mortality of HZ. In fact, administration of the vaccine to younger people may offer a good cost-benefit balance [28].

The availability of a specific anti-zoster vaccine could offer an important tool for reducing health problems and improve the overall quality of life in the elderly, as demonstrated by several studies [29-32]. A combined immunization against both varicella in childhood and HZ in adulthood in the developed world could improve the control of the virus [23]. The future challenge is represented by the prediction of how the decrease in VZV circulation will affect immunity among both vaccinated and unvaccinated individuals: varicella will continue to occur even in a highly immunized population, because of the ability of VZV to reactivate as zoster. Studies have predicted that there will be an increase in HZ incidence until the adult population becomes predominantly composed of individuals with vaccine-induced immunity who do not harbour wild-type VZV [22]. Moreover, VZV vaccination in children, without optimal coverage, can shift the disease to adults, leading to severe illness and a higher incidence of herpes zoster. Vaccination strategies will likely need to be adjusted as the epidemiology of VZV and risk factors continues to evolve [33]. Some risk factors may help to redirect preventive vaccination strategies. Hicks et al. demonstrated that, in a cohort of non-immunocompromised subjects, there was a strong association between the development of HZ and a family history of HZ, suggesting that these individuals were at high risk for HZ infection. These individuals could be the primary target for vaccination, thus, decreasing both their possibility of VZV infection and health care expenditures due to HZ morbidity [34].

Age can also be considered as a risk factor: active HZV vaccination of individuals > 50 years of age, simultaneously or in sequence with anti-influenza vaccination, has been demonstrated to be an effective, well-tolerated strategy [35,36]. Few studies have been conducted to investigate the social and epidemiological problems related to shingles disease or to the spread of immunization. In a recent global survey about awareness, knowledge, symptoms and treatment of HZ among 8,688 adults ≥ 50 years of age in 22 countries, there was wide variation in HZ awareness among the different areas and, almost, universally poor knowledge of the causes and symptoms of HZ were reported. Moreover, the majority of respondents were unaware of their risk of HZ. This survey suggests a population-wide effort to improve global awareness of HZ would be required for a successful vaccine initiative [37].

In the Netherlands, Opstelten et al. evaluated the determinants of non-compliance with HZ vaccination in community-dwelling elderly to whom a free HZ vaccination was offered simultaneously with the yearly influenza vaccination. In all, 690 patients (39%) were vaccinated against HZ, while 1,349 patients (76%) accepted influenza vaccination. Determinants of non-compliance with HZ vaccination included the perceived lack of recommendation by the GP, unwillingness to comply with the doctor's advice, perception of low risk for contracting HZ, perception of a short pain duration for HZ and the opinion that vaccinations weaken natural immunity [38].

In the present study we have compared attitude, knowledge and experience about VZV-associated diseases of individuals from different age groups in two different regions in Italy. In both regions, the majority of subjects had been affected by varicella infection, while a low percentage (5%) had been vaccinated against VZV. Regarding perception and availability of the HZ vaccination, in both regions, adults over the age of 21 seemed to be willing to accept this vaccination. This is probably due to greater knowledge of the infection/disease in this population and to a direct, or indirect, experience with HZ in the older age group.

Finally, limits and biases of this study should be mentioned. A cross-sectional study measures the prevalence of an outcome of interest in a certain population at a certain time point or over a short period. We have explored the acceptability of the HZ vaccination in people using face to face interviews, carried out by their pratictioners and in university staff. We randomly selected the interviewed population in two Italian regions, Lazio and Campania. Indeed, each of the associations identified in the current study must be interpreted with caution because our sample is not representative of the entire Italian population and study inclusion or exclusion could have been mediated by the interviewer during the face to face interview. A cross-sectional design may also make it difficult to establish the cause and the effect, i.e., for this study, the vaccine acceptability versus the knowledge of the disease and the vaccine acceptability versus the age group.

Moreover, it has to be remarked that our investigation didn't concern economical aspects of vaccination strategies. Therefore, the questionnaire do not comprise questions about willingness-to-pay of interviewed subjects.

## Conclusions

In this study compliance versus vaccination results were satisfactory and it is possible to assume that, with the upcoming availability of a HZ vaccine in Italy, adults will be the population favourably disposed towards in vaccination. However, the reported compliance must be considered only as an estimate and should be verified by larger public health interventions and broaden cost-effectiveness analyses.

## Competing interests

The authors declare that they have no competing interests.

## Authors' contributions

AP, VRS, MC and GL conceived of the study, and participated in its design and coordination. FF, EF and FG participated in the design of the study, performed the statistical analysis and helped to draft the manuscript. All authors read and approved the final manuscript.

## Pre-publication history

The pre-publication history for this paper can be accessed here:

http://www.biomedcentral.com/1471-2458/10/333/prepub

## Supplementary Material

Additional file 1 **Questionnaire**. A copy of the questionnaire employed in the study. Click here for file
