# Supplementary material for: Compliance with herpes zoster vaccination in young and adult individuals in two regions of Italy
Source: BMC Public Health. 2010 Jun 12;10:333. doi: 10.1186/1471-2458-10-333 (PMC2896358; doi:10.1186/1471-2458-10-333)
Supplement: Additional file 1 — Questionnaire. A copy of the questionnaire employed in the study. [file 1471-2458-10-333-S1.DOC]

**STUDY ON VACCINATION ACCEPTABILITY AGAINST**

**HERPES ZOSTER - "SHINGLES"**

The shingles (herpes zoster) is a common illness, especially among the elderly, known for causing burning pain; it can last for years and be disabling.

To cause herpes zoster is a reactivation varicella virus that remains latent in nerve ganglia in people who infected in childhood and partially immunized, but it reasserts at an impairment immune system.

It is estimated that every year, there are about 1.5 million new cases of Herpes Zoster in Europe. In Italy there are about 250-300 thousand new cases each year.

There is no still effective therapy to prevent herpes zoster and the vaccine may thus represent a turning point.

QUESTIONNAIRE

Date compilation |__|__| |__|__| |__|__|

YY mm DD

1. You refuse to answer the questionnaire because ……………………………………………………………………………………

…………………………………………………………………………………………………………………………………………………………………………………………………………………………………………………………………………………………………………………………………………

Thank you for agreeing to participate in this study. Now you will answer some questions about your life. The information contained in the questionnaire will be kept confidential as required by law.

**1. Sex** 🞏 Male 🞏 Female 2. Age|__|__|

**3. Nationality** __________________________ **4.** **Residence district**|__|__|

#### 5. Graduation

🞏 Anyone (illiterate)

🞏 Primary school

🞏 Middle school

🞏 High school

🞏 University degree

**11. Do you Know disease called "Varicella"?**

🞏 no

🞏 yes

**12. Were you ill with varicella in the past?**

🞏 no

🞏 yes

**13. If so, at what age?**

**14. Have you been vaccinated for varicella?**

🞏 no

🞏 yes

**15. If so, at what age?**

**16. Have you contact with a person with varicella?**

🞏 no

🞏 yes

**17. Do you know disease called "shingles" (herpes zoster)?**

🞏 no

🞏 yes

**18. Were you ill with shingles in the past?**

🞏 no

🞏 yes

**19. If so, at what age?**

**20. Do you knows someone who had the "shingles" (herpes zoster)?**

🞏 no

🞏 yes

**21. If there was a vaccine against shingles (herpes zoster) do you vaccinate?**

🞏 no

🞏 yes
